# Radiofrequency volumetric thermal ablation of fibroids: a prospective, clinical analysis of two years’ outcome from the Halt trial

**DOI:** 10.1186/1477-7525-11-139

**Published:** 2013-08-13

**Authors:** Richard S Guido, James A Macer, Karen Abbott, Janice L Falls, Ian B Tilley, Scott G Chudnoff

**Affiliations:** 1 Department of Obstetrics, Gynecology and Reproductive Sciences, Division of Gynecologic Specialties, University of Pittsburgh Medical Center, Magee-Women’s Research Institute, Pittsburgh, PA, USA; 2 Department of Obstetrics and Gynecology, Huntington Hospital, Pasadena, CA, USA; 3 Athena Gynecology Medical Group, Reno, NV, USA; 4 Department of Obstetrics and Gynecology and Women’s Health, Montefiore Medical Center, Albert Einstein College of Medicine, Bronx, NY, USA; 5 Department of Clinical Obstetrics and Gynecology, Los Angeles County and University of Southern California Medical Center, Los Angeles, USA

**Keywords:** Radiofrequency ablation, Ultrasound, Laparoscopic ultrasound, Fibroid, Myoma, Quality of life

## Abstract

**Background:**

Although most myomas are asymptomatic, quality of life is compromised for many women with uterine fibroid disease. Twelve-month outcomes from the Halt Trial have been reported in the literature. Here we analyze the clinical success of radiofrequency volumetric thermal ablation (RFVTA) of symptomatic uterine fibroids at two years of follow up.

**Methods:**

Prospective, multicenter, outpatient interventional clinical trial of fibroid treatment by RFVTA in 124 premenopausal women (mean age, 42.4 ± 4.4 years) with symptomatic uterine fibroids and objectively confirmed heavy menstrual bleeding (≥160 to ≤500 mL).

Outcome measures included: subject responses to validated questionnaires, treatment-emergent adverse events, and surgical re-intervention for fibroids at 24 months postprocedure. Continuous and categorical variables were summarized using descriptive statistics and means and percentages. Comparisons between visits were based on t-tests using repeated measures models. P-values < 0.05, adjusted for multiplicity, were statistically significant.

**Results:**

One hundred twelve subjects were followed through 24 months. Change in symptom severity from baseline was –35.7 (95% CI, –40.1 to –31.4; p<.001). Change in health-related quality of life (HRQL) was 40.9 (95% CI, 36.2 to 45.6; p < .001). HRQL subscores also improved significantly from baseline to 24 months in all categories (concern, activities, energy/mood, control, self-consciousness, and sexual function) [p<.001]. Six patients underwent surgical re-intervention for fibroid-related bleeding between 12 and 24 months providing a re-intervention rate of 4.8% (6/124).

**Conclusion:**

Radiofrequency volumetric thermal ablation of myomas significantly reduces symptom severity and improves quality of life with low surgical re-intervention through 24 months of follow up.

**Trial registration:**

ClinicalTrials.gov: NCT00874029

## Background

Although most uterine fibroids are asymptomatic, heavy menstrual bleeding presents as the most common complaint among women who undergo myomectomy [1]. For premenopausal women, average menstrual blood loss is 40 ± 20 mL [2] with monthly bleeding > 60 mL possibly leading to anemia from iron deficiency as well as affecting quality of life [3]. Chudnoff et al reported efficacy, safety and re-intervention rates up to 12 months after radiofrequency volumetric thermal ablation (RFVTA) in 135 women, who had participated in a prospective multicenter interventional clinical trial for the treatment of uterine myomas and heavy menstrual bleeding (≥ 160 mL to ≤ 500 mL) [ClinicalTrials.gov Identifier: NCT00874029] [4]. The RFVTA system delivers monopolar radiofrequency energy to tissue through a disposable electrosurgical handpiece; the handpiece is attached to the control unit and is inserted percutaneously into the target fibroid tissue [5].

The continued analysis of the subjects’ wellbeing beyond the initial study period of 12 months provides a realistic assessment of the medium-term efficacy of the RFVTA treatment. Our objectives were analyses of qualitative clinical outcomes, as evidenced by subject responses to validated questionnaires [6,7], as well as the incidence of device- and/or procedure-related adverse events and surgical re-intervention for fibroid treatment among those subjects followed to 24 months post RFVTA with the Acessa System (Halt Medical, Inc., Brentwood, California USA).

## Materials and methods

For this prospective, interventional clinical trial [ClinicalTrials.gov Identifier: NCT00874029], in which subjects served as their own controls, we recruited and enrolled premenopausal women with ≤ 6 treatable fibroids with no single fibroid exceeding 7 cm in any diameter; these parameters were confirmed by transvaginal ultrasound. The subjects’ primary complaint was heavy cyclic menstrual bleeding (≥ 160 mL to ≤ 500 mL), which was confirmed by alkaline hematin analysis. For the clinical trial, the U.S. Food and Drug Administration required menstrual blood loss assessment through 12 months of follow up and suggested quality-of-life assessments thereafter. Recruitment started in February 2009 and enrollment of the last patient was in February of 2011. All subjects signed informed consent and the 11 clinical sites in the United States (N = 9) and Latin America (N = 2) [Appendix A] obtained local institutional review board (IRB) or independent ethics committee (IEC) approval of the protocol. The study continues to be conducted in accordance with the general ethical principles outlined in the Declaration of Helsinki and applicable local or international regulations concerning the rights and welfare of human subjects participating in medical research.

Inclusion criteria for enrolled subjects were premenopausal women of at least 25 years of age, with symptomatic uterine fibroids imaged by transvaginal ultrasound and magnetic resonance imaging, having a uterine size of ≤ 14 weeks as determined by pelvic examination, a total fibroid volume of ≤ 300 cm^3^, a minimum of a three-month history of heavy menstrual bleeding within 6 months of enrollment and desiring uterine conservation . Laboratory inclusion criteria were a normal coagulation profile, a normal Papanicolaou test result in the last year, and a hemoglobin level of ≥ 10.0 g/dL at the time of treatment. Exclusion criteria were radiologic evidence by magnetic resonance imaging of adenomyosis, pedunculated intracavitary or subserosal fibroids (presence of types I and II submucous fibroids were allowed); history of pelvic malignancy or cervical dysplasia; prior treatment or removal of fibroids; and women desiring future childbearing. Any contraindications to abdominal surgery or to anesthesia excluded enrollment. The treatment was free for all study participants.

Radiofrequency volumetric thermal ablation was performed with the Acessa System, which is comprised of a dual-function radiofrequency generator, a disposable 3.4-mm handpiece with a deployable seven-needle electrode array and controls for inputting data and modifying generator parameters; two disposable dispersive electrode pads, which were placed on each thigh; extension cables; and a foot pedal. A 10-mm or 12-mm laparoscope was inserted through a 10- or 12-mm umbilical trocar, and laparoscopic ultrasound through a separate 10- or 12-mm trocar (Aloka, Wallingford, CT USA; BK Medical, Peabody, MA USA) permitted mapping of the uterus and identification of the location, size, and number of all fibroids. The handpiece was inserted percutaneously (without a trocar) and into the fibroid using ultrasound guidance and the ablation procedure was carried out. Details of the procedure and technology have been presented elsewhere [4,8]. Patients were discharged on the day of treatment after standard postoperative care with instructions to return to work and to normal activities as they felt able and to refrain from sexual activity for 4–6 weeks. Ibuprofen, naproxen or celecoxib were prescribed for pain on an as-needed basis. At each of the follow-up visits (3, 6, 12, and 24 months post procedure), subjects provided written responses to the validated Uterine Fibroid Symptom and Quality-of-Life questionnaire (UFS-QOL) [6] and responded to general health and outcome assessments as measured by the EuroQol Health State Index, (EQ-5D) [7].

Continuous variables for the analyses performed for this report were summarized using descriptive statistics, whereas categorical variables were summarized in terms of frequencies and percentages. Pairwise comparisons between the baseline visit and the 3, 6, 12, and 24 month follow-up visits and pairwise comparisons between each of the follow-up visits with regard to each UFS-QOL scale and subscale and the EQ-5D health state scale were based on t-tests using repeated measures models, where the scores at each visit were the dependent variables. Because there were 10 possible pairwise comparisons between visits for each scale or subscale, p-values were adjusted for multiplicity using the Šidák correction; the adjusted p-values are presented. P-values of < 0.05 were considered statistically significant.

## Results

**Figure 1:**
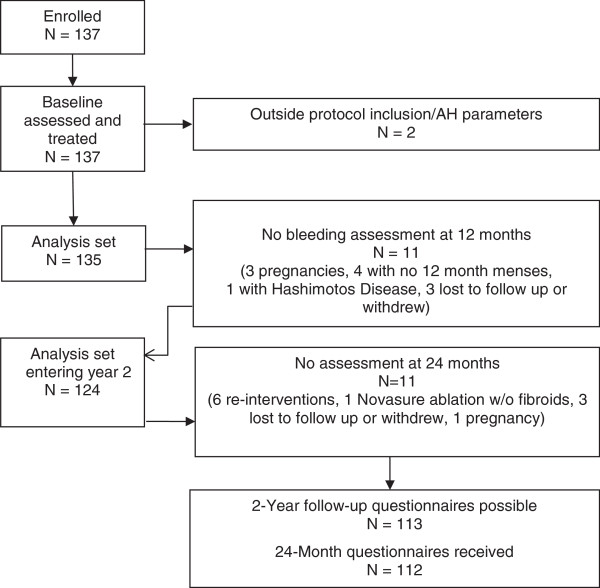
Status of study subjects at 24 months post procedure.

The progression of subjects through their 24-month visits following RFVTA is presented in Figure 1. Of the 135 women satisfying all inclusion criteria and treated at baseline, 11 subjects had interfering circumstances unrelated to the procedure (pregnancy, lack of menses, and Hashimoto’s Disease) that could have influenced bleeding assessments positively or negatively. Of the 124 subjects continuing past the 12-month follow up, six sought surgical re-interventions, one subject became pregnant, one chose Novasure ablation as diagnostic hysteroscopy revealed no myomas, and three subjects were lost to follow up or withdrew from the trial. Of the outstanding 113 Uterine Fibroid Symptom and Quality-of-Life questionnaires, the 11 sites received 112 completed questionnaires. Demographics of those 124 subjects entering the second year of the study are presented in Table 1.

**Table 1:** Demographics characteristics of subjects entering 12 months follow up (n = 124)

**Variable**	**Statistic/Response**^**a**^	**All sites (n = 124)**
Age (years)	Mean (SD)	42.4 (4.4)
	Median	43
	Range	31 – 52
Race	White or Caucasian	58 (46.8%)
	Black or African American	41 (33.1%)
	Asian	2 (1.6%)
	Other ^b^	23 (18.5%)
Ethnicity	Hispanic or Latino	56 (45.2%)
	Not Hispanic or Latino	68 (54.8%)
Smoking History	Current	25 (20.2%)
	Past	21 (16.9%)
	Never	78 (62.9%)
Height (cm)	Mean (SD)	162.5 (8.1)
	Median	162.6
	Range	137.2 – 180.3
Weight (kg)	Mean (SD)	80.9 (19.4)
	Median	79.2
	Range	49.0 – 147.4
BMI	Mean (SD)	30.5 (6.2)
	Median	29.2
	Range	19.8 – 47.3

^a^*SD* Standard deviation.

^b^ Other, Hispanic, hispanic indigenous, or Caribbean.

**Figure 2:**
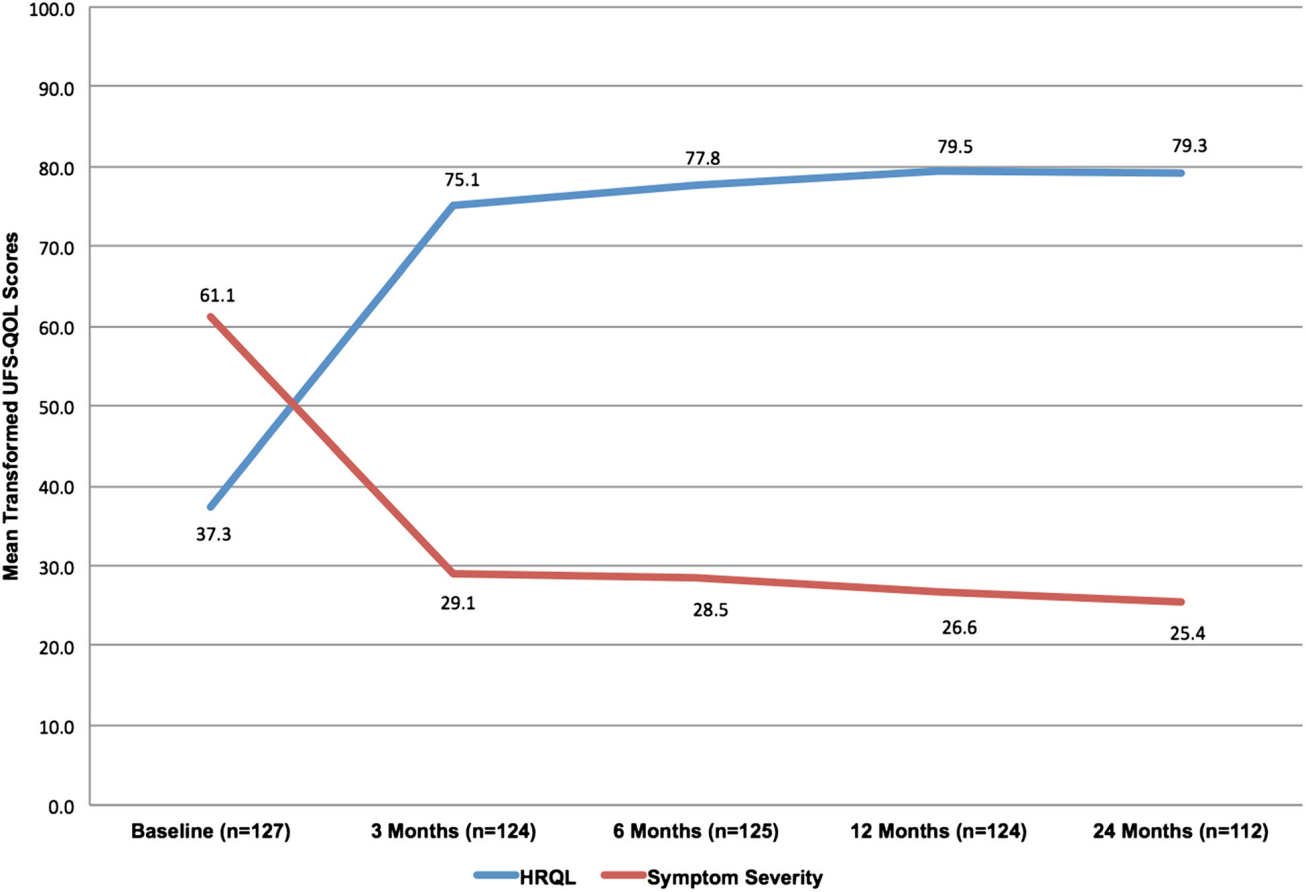
Mean transformed scores of patient-reported symptom severity and health-related quality of life (UFS-QOL).

As shown in Figure 2, patient-reported symptom severity decreased (improved) most readily from baseline (61.1 ± 18.6) in the first three months of follow up. Symptom severity scores at 3 months (29.1 ± 18.9), 6 months (28.5 ± 19.3), 12 months (26.6 ± 19.0), and 24 months (25.4 ± 20.6) were similar. The change from the mean baseline value to that at 24 months for the 112 subjects with baseline and 24-month symptom severity scores was –35.7 (95% CI, –40.1 to –31.4).

Health-related quality of life also improved most readily from baseline (37.3 ± 19.1) to 3 months (75.1 ± 22.1). Successive scores were similar to the 3-month value: 6 months (77.8 ± 20.2), 12 months (79.5 ± 20.6), and 24 months (79.3 ± 21.7). The change at 24 months for the 112 subjects with baseline and 24-month HRQL scores was 40.9 (95% CI, 36.2 to 45.6).

**Figure 3:**
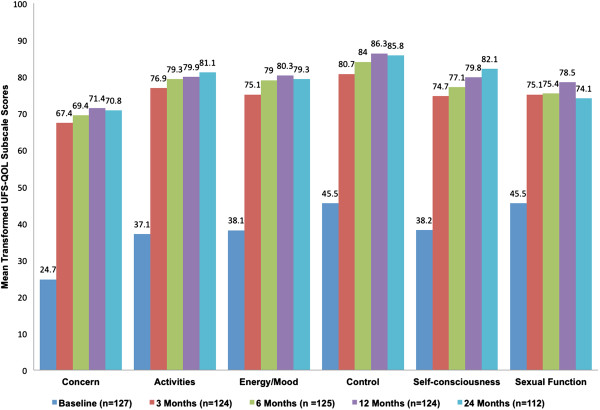
Mean transformed scores of patient-reported UFS-QOL subscale scores over time.

Patient-reported UFS-QOL subscale scores also improved most readily in the first 3 months of follow up (Figure 3). Improvement from baseline to 24 months for each of the subscales is summarized in Table 2. Concern showed the most improvement (Δ 45.6) and sexual function, although statistically improved, showed the least improvement (Δ 29.2) over the study period. These changes were based on those 112 subjects who had both baseline and 24-month scores.

**Table 2:** Improvements in UFS-QOL subscale scores from baseline to 24 months

**Subscale**	**Baseline**	**24 months**	**Change in score**	**95% confidence interval**
Concern	24.7 ± 20.7	70.8 ± 28.6	45.6	39.9, 51.3
Activities	37.1 ± 24.1	81.1 ± 24.2	41.9	37.5, 48.2
Energy/Mood	38.1 ± 21.8	79.3 ± 22.9	39.6	34.6, 44.6
Control	45.5 ± 24.9	85.8 ± 22.1	39.1	33.4, 44.9
Self-consciousness	38.2 ± 28.3	82.1 ± 21.1	42.0	36.3, 47.7
Sexual function	45.5 ± 29.8	74.1 ± 29.1	29.2	22.7, 35.8

**Figure 4:**
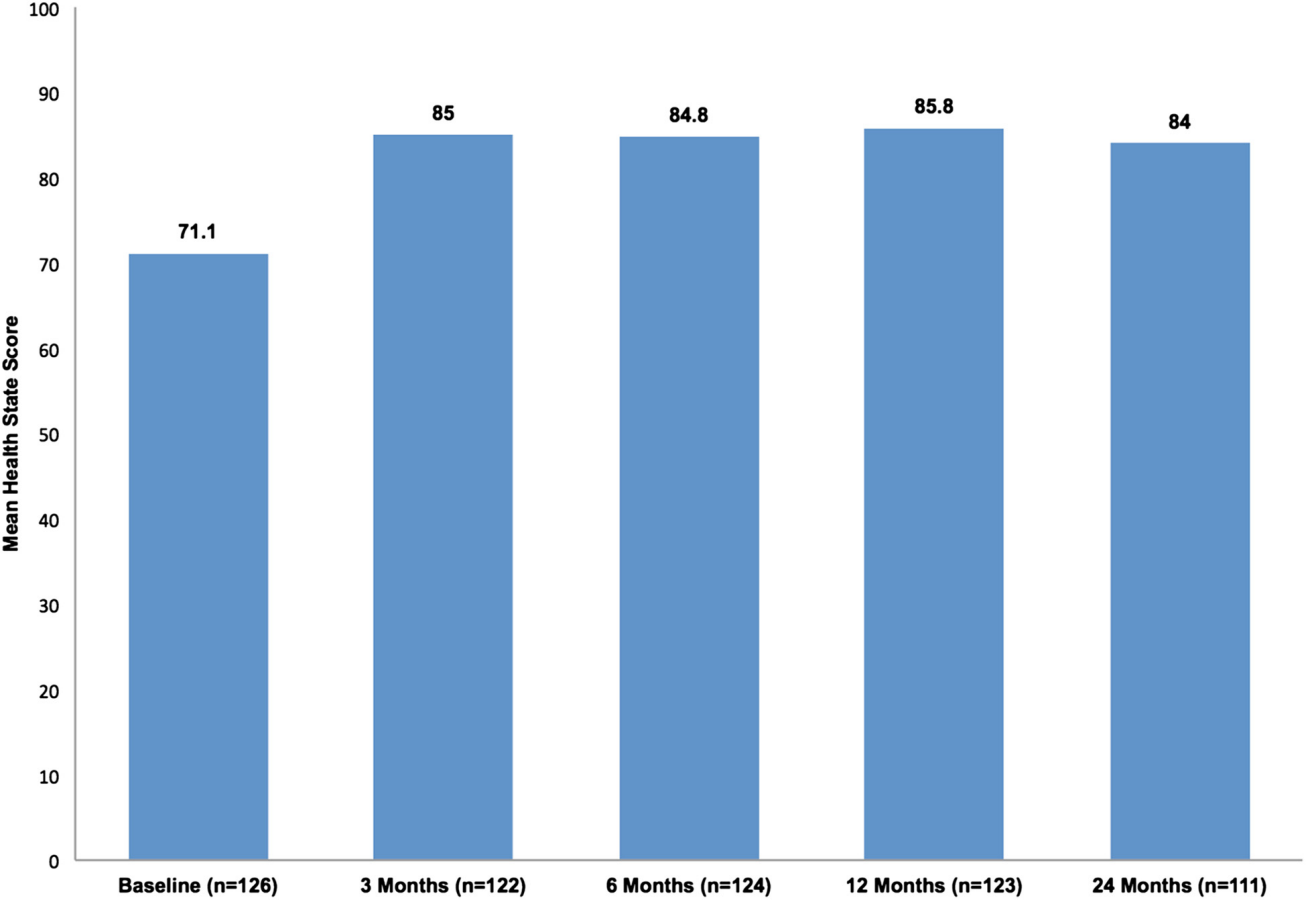
Mean health state (EQ-5D) scores over time.

Mean health state scores (EQ-5D) improved from baseline to 3 months and then changed slightly over time from 85.0 to 84.0 (Figure 4). There was a significant improvement in the mean health state score between baseline and 3 months after treatment (p < .001). Measurements at subsequent intervals showed no continued improvement, but remained statistically improved over baseline.

There was one serious adverse event, which occurred between 12 and 24 months and was possibly related to the procedure. One subject became pregnant and delivered a healthy, full-term baby by Cesarean section. However, during the Cesarean section, the subject lost 1400–1500 mL of blood. Approximately 48 hours later, she experienced abdominal pain with additional blood loss and tissue expulsion. Preliminary pathology indicated degenerative fibroid tissue. The patient received 6 units of blood altogether and was discharged from the hospital with oral iron therapy for her anemia.

Six patients (6/124, 4.8%) underwent surgical re-intervention for fibroid-related bleeding between 12 and 24 months (Table 3): 4 hysterectomies and 2 hysteroscopic myomectomies. Follow-up pathology revealed multiple small fibroids with adenomyosis in four cases (patients 1, 3, 4, and 6), and a possible polyp (patient 5). Pathology studies were not available for Patient 2.

**Table 3:** Surgical re-interventions (6/124, 4.8%) between 12 and 24 months post procedure

**Pt**	**Treated fibroids**^**a**^	**Symp severity**^**b**^		**HRQL**^**c**^		**Reintervention**	**Pathology**
		**Baseline**	**12 mo**	**Baseline**	**12 mo**		
**1**	6 Subserosals 5.6; 4.4; 1.5; 3.6; 1.9; 3.0	53.1	28.1	46.6	89.7	Hysterectomy at 16.5 months	Multiple myomas ranging from 0.4 to 4.7 cm; focally irregular endo-myometrial junction
	2 Intramurals 4.8: 4.7						
	1 Subserosal/Intramural 2.7						
**2**	4 Intramurals 5.2; 1.8; 1.1; 1.8	68.8	28.1	10.3	82.8	Hysteroscopic myomectomy by resection at 15 months	No pathology
**3**	3 Subserosals 5.0; 1.3; 1.9	78.1	43.8	20.7	51.7	Hysterectomy at 14 months	Multiple myomas ranging from 0.6 to 3.4 cm; focal adenomyosis
	1 Intramural 1.8						
**4**	1 Intramural 2.0	62.5	28.1	10.3	60.3	Supracervical hysterectomy at 23 months	Only morcellated tissue available. Findings: adenomyosis, leiomyomata, proliferative endometrium
	1 Submucosal 2.0 1						
	Undefined 1.9						
**5**	5 Intramurals 2.7; 2.7; 2.6; 8.5; 6.7	75.0	56.3	17.2	28.4	Hysteroscopic myomectomy by resection at 16 months	Focal degenerative changes and features suggestive of polyp
	1 Undefined 3.2						
**6**	1 Intramural 2.0	53.1	62.5	37.1	43.1	Hysterectomy at 23.5 months	Adenomyosis; multiple myomas ranging in size from 0.4 cm to 1.2 cm
	1 Subserosal 2.0						

^a^ Information includes the number of each type of treated fibroid and the maximum diameter (cm) of each fibroid.

^b^ Mean transformed symptom severity scores.

^c^ Mean transformed health-related quality-of-life scores.

## Discussion

The subject device (the Acessa System, Halt Medical, Inc., Brentwood, California USA) and the fibroid ablation procedure (RFVTA) were developed and refined by Lee and others specifically for fibroids [5,9]. Despite resolution of fibroid symptoms achieved in his early trials using other radiofrequency ablation systems, Lee found the characteristic bending of the needles an obstacle to accurate and dependable fibroid ablation. The current Acessa System and RFVTA procedure showed promise in feasibility studies carried out in Latin America [8,10] and proved effective and safe during the first 12 months of follow up in the FDA-approved prospective interventional clinical trial [4].

This study describes the continued benefits from 12 months [4] through 24 months of follow up; general and disease-specific quality-of-life outcomes are described. The UFS-QOL and EQ-5D scales have been validated and reported as appropriate for use with long-term follow up of patients with symptomatic uterine fibroids [11]. Coyne et al examined the validity, reliability and responsiveness of the UFS-QOL scale among women with symptomatic fibroids through 12 months of follow up after treatment and among controls (premenopausal women without fibroids) [12]. The patients in our study showed significant improvement in their symptom severity and health-related quality of life from baseline to 3 months post treatment. This was sustained over 2 years, accompanied by a low rate of re-intervention (4.8%). The 3-month outcomes appear to be predictive of long-term (≥ 2 years) results. Some patients, who did not improve at 3 months, showed improvement at later follow-up periods. Continued follow up to 36 months is planned. Researchers of other fibroid treatments have reported similar improvements in symptom severity and quality of life but with re-intervention rates after two-to-three years nearing 26% [10,13,14].

The introduction of new technologies in gynecology has increased women’s options for the treatment of benign gynecologic conditions. These include the use of global endometrial ablation as an alternative to hysterectomies, especially for those women with abnormal bleeding and adenomyosis, and the use of uterine fibroid embolization for the treatment of fibroids. However, it is important to track the outcomes of new procedures to confirm the durability of the treatment.

## Conclusions

Radiofrequency volumetric thermal ablation broadly met the needs of the study patients. The low adverse event and re-intervention rates through 24 months are positive outcomes for patient wellbeing and demonstrate that the improvement in symptoms and the increase in the patients’ quality of life are durable for at least a period of 2 years.

## Appendix A

### The Halt Study Group

Scott G. Chudnoff, MD, MS

Department of Obstetrics & Gynecology and Women’s Health, Montefiore Medical Center, Einstein and Moses Divisions, Albert Einstein College of Medicine, Bronx, NY, USA

Erika Banks, MD

Department of Obstetrics and Gynecology and Women’s Health, Montefiore Medical Center, Albert Einstein College of Medicine, Bronx, NY USA

Micah Harris, MD

Women’s Health Research, Phoenix, AZ, USA

José Gerardo Garza Leal, MD

Hospital Universitario Nuevo Leon, Monterrey, Nuevo Leon, Mexico

Rodolfo Robles Pemueller, MD

Hospital Universitario Esperanza, Universidad Francisco Marroquin, Guatemala City, Guatemala

Karen Abbott, MD

Athena Gynecology Medical Group, Reno, NV, USA

Jay M. Berman, MD

Department of Obstetrics and Gynecology, Division of Gynecology, Wayne State University School of Medicine, Detroit, MI, USA

David J. Levine, MD

St. John’s Mercy Hospital, St. Louis, MO, USA

Donald I. Galen, MD

Reproductive Science Center of the San Francisco Bay Area, San Ramon, CA, USA

James A. Macer, MD

Pasadena Premier Women’s Health, Pasadena, CA, USA

Janice L. Falls, MD

Department of Obstetrics & Gynecology and Women’s Health, Montefiore Medical Center, Einstein and Moses Divisions, Albert Einstein College of Medicine, Bronx, NY, USA

Ian B. Tilley, MD

Department of Clinical Obstetrics and Gynecology, Los Angeles County and University of Southern California Medical Center, Los Angeles, CA, USA

Richard S. Guido, MD

Department of Obstetrics, Gynecology and Reproductive Sciences, Division of Gynecologic Specialties, University of Pittsburgh Medical School, Magee-Women’s Research Institute, Pittsburgh, PA, USA

## Abbreviations

RFVTA: Radiofrequency volumetric thermal ablation; HRQL: Health-related quality of life; EQ-5D: EuroQol health state index; IRB: Institutional review board; IEC: Independent ethics committee; CT: Connecticut; MA: Massachusetts; USA: United States of America; UFS-QOL: Uterine fibroid symptom and quality of life.

## Competing interests

The authors received financial support from the study sponsor for supplies and use of hospital and administrative services for the study. RSG has been a consultant, assisting in the development of educational materials for the study sponsor. The authors have no other disclosures.

## Authors’ contributions

RSG participated in the design of the study as a principal investigator and surgeon, reported data, and helped draft the manuscript. JAM participated as a principal investigator and surgeon, reported data, and reviewed all drafts of the manuscript. KA participated as a principal investigator and surgeon, reported data, and reviewed all drafts of the manuscript. JLF participated as a principal investigator and surgeon, reported data, and reviewed all drafts of the manuscript. IBT participated as a principal investigator and surgeon, reported data, and reviewed all drafts of the manuscript. SGC participated in the design of the study as principal investigator and surgeon, reviewed and critiqued the statistical analyses, reported data, and reviewed all drafts of the manuscript. All authors read and approved the final manuscript.

## Authors’ information

All authors are experienced, board-certified, minimally invasive gynecologic surgeons. In addition, SGC has an advanced degree in biostatistics.
